# Dynamic peripheral visual performance relates to alpha activity in soccer players

**DOI:** 10.3389/fnhum.2014.00913

**Published:** 2014-11-11

**Authors:** Wenya Nan, Daria Migotina, Feng Wan, Chin Ian Lou, João Rodrigues, João Semedo, Mang I Vai, Jose Gomes Pereira, Fernando Melicio, Agostinho C. Da Rosa

**Affiliations:** ^1^Department of Electrical and Computer Engineering, Faculty of Science and Technology, University of MacauMacau, China; ^2^Biomedical Engineering and Evolutionary Systems Lab, Systems and Robotics InstituteLisbon, Portugal; ^3^Academia do Sporting Club de Portugal and Faculdade de Motricidade Humana, University of LisbonLisbon, Portugal; ^4^Department of Electronics and Telecommunications and of Computers Engineering, Instituto Superior de Engenharia de Lisboa, IPLLisbon, Portugal; ^5^Department of BioEngineering, Instituto Superior Tecnico, University of LisbonLisbon, Portugal

**Keywords:** dynamic peripheral vision, relationship, alpha activity, soccer player, individual alpha band

## Abstract

Many studies have demonstrated the relationship between the alpha activity and the central visual ability, in which the visual ability is usually assessed through static stimuli. Besides static circumstance, however in the real environment there are often dynamic changes and the peripheral visual ability in a dynamic environment (i.e., dynamic peripheral visual ability) is important for all people. So far, no work has reported whether there is a relationship between the dynamic peripheral visual ability and the alpha activity. Thus, the objective of this study was to investigate their relationship. Sixty-two soccer players performed a newly designed peripheral vision task in which the visual stimuli were dynamic, while their EEG signals were recorded from Cz, O1, and O2 locations. The relationship between the dynamic peripheral visual performance and the alpha activity was examined by the percentage-bend correlation test. The results indicated no significant correlation between the dynamic peripheral visual performance and the alpha amplitudes in the eyes-open and eyes-closed resting condition. However, it was not the case for the alpha activity during the peripheral vision task: the dynamic peripheral visual performance showed significant positive inter-individual correlations with the amplitudes in the alpha band (8–12 Hz) and the individual alpha band (IAB) *during* the peripheral vision task. A potential application of this finding is to improve the dynamic peripheral visual performance by up-regulating alpha activity using neuromodulation techniques.

## Introduction

Human visual system is composed of central vision and peripheral vision. As an important constituent part of vision, peripheral vision occurs outside the central field of view and is responsible for the peripheral visual information collection. According to the biological constitution of human eyes, it is well-known that retina is made up by two types of photoreceptor cells, namely rod cells and cone cells. Cone cells are mostly concentrated in the central area of the retina and less densely populated in the periphery, while rod cells are commonly distributed in the outer edges of the retina and peripheral vision mainly employs rod cells. When an object exceeds the central visual field, humans have to make saccadic eye movements to search the object, which will bring parts of the object into the central vision. The orientation and span of eye movements use the visual information from peripheral vision. Peripheral vision provides plentiful visual information outside the central visual field and is important for feature recognition and object identification because it directs eye movements in neutral search tasks (Torralba et al., 2006) and provides the visual information as important triggers for saccades (Luo et al., 2008).

Particularly, the peripheral vision in the dynamic visual environment (i.e., dynamic peripheral vision) is crucial (e.g., driving, walking, playing, etc.). For example, drivers need good peripheral vision since the driving safety depends strongly on the detection ability of moving cars and other objects in the peripheral visual field, as well as the ability to react to these visual stimuli in a timely manner (Hu et al., 2014). Team sports practitioners need high peripheral visual ability to sense the sports environment, as the peripheral vision facilitates motion detection (Knudson and Kluka, 1997) and its reaction time is faster than that of saccadic vision. Moreover, good peripheral vision is beneficial for either monitoring surroundings or maintaining steady balance in team sports. For instance, when a player wants to pass the soccer ball to his teammate, he should not look at his teammate directly and make a heel-dragging decision. Otherwise, he may lose the control of the ball as the defender would identify and prevent the passing through the detection of the opponent's eye gaze. Therefore, the player has to employ his peripheral vision to gather information from the sports environment and keep focused without revealing his intention so as to avoid defending actions by his opponents. If a player has better peripheral vision ability, he can notice his teammate earlier and make a better pass with a higher success rate.

In the literature, an increasing number of studies have demonstrated that the EEG alpha activity is closely linked to a variety of cognitive functions such as attention, memory, perception, and exploratory behavior. For instance, in a number of selective attention tasks, the increase of alpha power appears over cortical areas responsible for processing potentially distracting information, and these attention-related sustained focal increases in alpha power occur prior to the arrival of an anticipated stimulus (Foxe and Snyder, 2011). Moreover, alpha amplitudes increase when subjects shift their attention inwards (Ray and Cole, 1985; Cooper et al., 2003) and decrease if a stimulus is expected (Worden et al., 2000; Sauseng et al., 2005; Thut et al., 2006). Regarding the semantic memory, the resting alpha power is positively associated with memory performance, whereas during actual processing of the task, small alpha power is related to good performance (Klimesch et al., 2006). Contrary to memory, good central vision perception performance is associated with low resting alpha power (Hanslmayr et al., 2005, 2007) and low prestimulus alpha power (Ergenoglu et al., 2004; Hanslmayr et al., 2005, 2007; van Dijk et al., 2008; Mathewson et al., 2009; Wyart and Tallon-Baudry, 2009).

In the above studies, different research objectives led to different designs of the visual tasks. In order to investigate the preparatory mechanisms of attention biasing, the visual attention task employs S1-S2 cuing paradigm in which a symbolic cue actively instructs the subject in an unpredictable manner to switch attention between tasks. Usually, a symbolic cue (stimulus 1-S1) such as an arrow, sound, or word informs of an upcoming task or indicates a specific location, modality, or feature for a subsequent stimulus (stimulus 2-S2) (Foxe and Snyder, 2011). In the investigation of the central vision perception performance, the stimuli are usually static and presented in the central visual field very briefly, and the subjects are instructed to discriminate the stimuli as quick as possible (e.g., Hanslmayr et al., 2005, 2007; van Dijk et al., 2008).

Different from central vision perception, peripheral vision is to identify and perceive the changes that occur in the circumjacent environment. So far, the peripheral vision measurements based on perception tasks in the major existing work are relatively static, that is, subjects only need to maintain their focus on a fixed point and report whether they can perceive the target appearance in the periphery. However, in a real visual environment, especially in the team sports involving a ball, the environment is usually dynamic rather than static, and the speed of movement is inconstant (such as opponents, teammates, and the ball). In order to evaluate the dynamic peripheral visual ability, Rodrigues et al. (2012) designed a new peripheral visual task in which the stimuli objects were dynamic. Five stimuli objects were presented at the four corners and the center of a LCD screen. The central object kept random movement within a small range and the appearance of the five objects had dynamic changes over stimuli trials. The exposure time of stimulus progressively decreased along trials. The subjects were required to track the central moving object binocularly with a mouse pointer and keep their sight on the central object, meanwhile perceive the four corner objects with their peripheral vision. The subjects were instructed to press the mouse as fast as possible when they perceived a target stimulus (three out of five objects were exactly the same). In such a way, a dynamic visual environment was mimicked and the dynamic peripheral visual ability was measured. Moreover, it was found that the sports performance in soccer players had significant positive correlation with the dynamic peripheral visual ability assessed by this visual task (Rodrigues et al., 2012).

A number of studies have already reported a close relationship between alpha activity and visual perception, however the visual perception is usually assessed by static stimuli. The real environment we live has often dynamic changes and therefore the peripheral visual ability in a dynamic environment is imperative. So far, no study has investigated the relationship between alpha activity and the dynamic peripheral visual ability. Therefore, the objective of this work was to explore this relationship. Considering the importance of dynamic peripheral visual ability in athletes, 62 soccer players were recruited as the subjects and their dynamic peripheral visual performances were assessed through the newly designed peripheral vision task (Rodrigues et al., 2012). We examined whether the dynamic peripheral visual performance was correlated with the alpha activity in the eyes-open and eyes-closed resting condition as well as during the peripheral vision task.

## Materials and methods

### Participants

Sixty-two soccer players [aged 14–19 years: mean = 16.44, standard deviation (SD) = 1.51] with normal vision took part in the experiment. Informed written consent was obtained from all participants before experiment. The protocol was approved by the local ethics committee, and each subject gave written informed consent before the experiment, according to the Declaration of Helsinki. One subject was excluded in the data analysis due to bad EEG signal quality.

### Signal recording

EEG was recorded from Cz, O1, and O2 according to the international 10–20 system. The reference was the average of both mastoids and the ground was placed on the forehead. To detect eyes movement, two signals of electrooculogram (EOG) were recorded and their difference was calculated. One electrode was placed 0.5 cm below the outer canthus of one eye, and another electrode was placed 0.5 cm above the outer canthus of the other eye. This particular configuration captured both horizontal and vertical eye movements in order to detect the responses to the stimuli with peripheral vision or eye scanning. The EEG and EOG signals were amplified by an amplifier (Vertex 823 from Meditron Electomedicina Ltda, SP, Brazil) with a sampling rate of 256 Hz and recorded by Somnium system (Cognitron, SP, Brazil). The impedance was kept below 10 kΩ for all electrodes. Before the peripheral vision measurement, two 1-min EEG epochs under the resting conditions were recorded, one with eyes open and another with eyes closed. Then, the participants performed the peripheral vision task.

### Peripheral vision assessment

**Figure 1:**
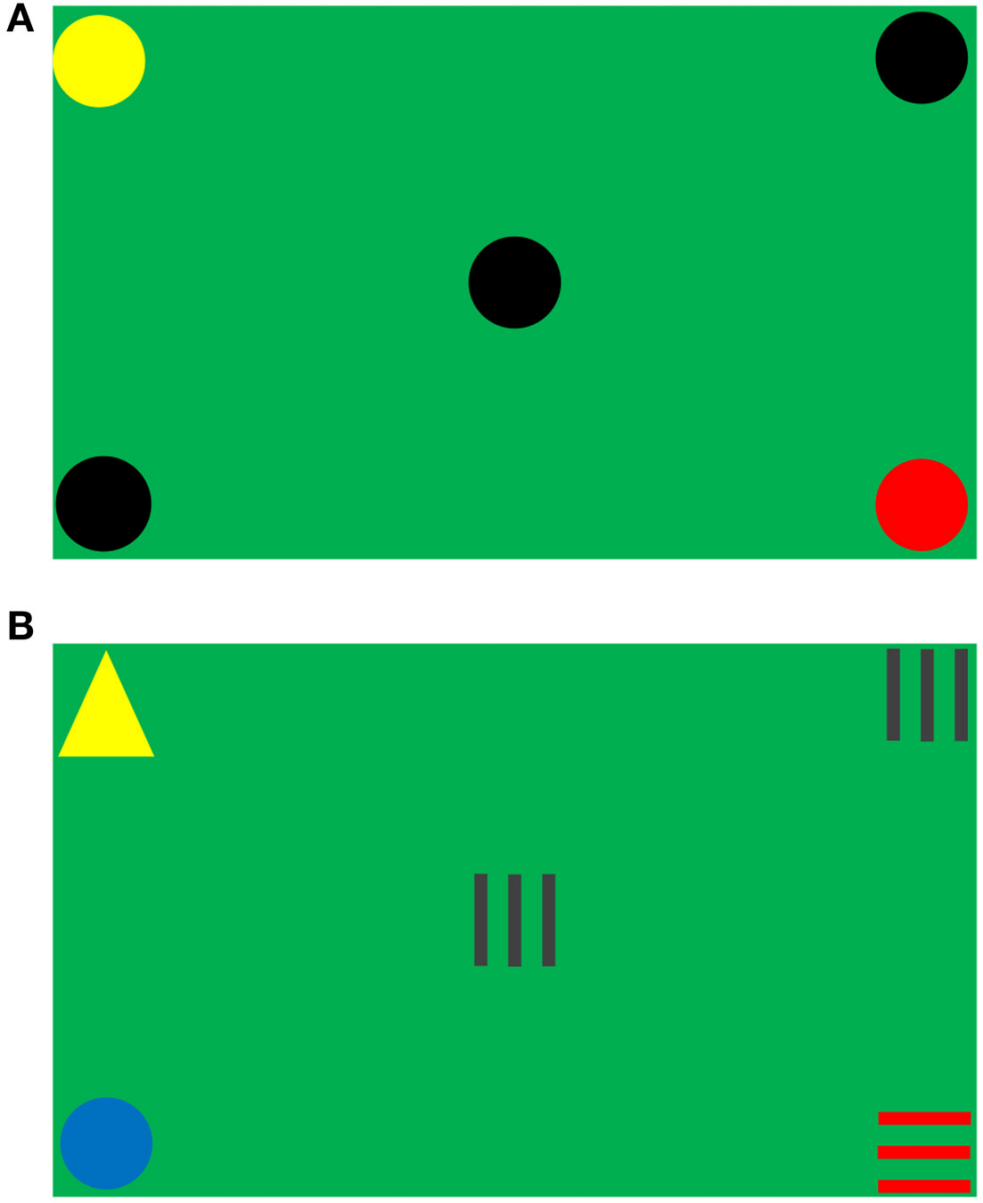
**Test screen. (A)** Target pattern in Session 1. **(B)** Non-target pattern in Session 2.

The visual task was designed to measure dynamic peripheral visual ability (Rodrigues et al., 2012). A diagonal size of 102 cm LCD screen with a resolution of 1920 × 1080 dots displayed the stimuli objects, and the refresh rate of LCD screen was 100 Hz. Figure 1 shows the test screen of the experiment, the background color simulated the soccer field environment and five stimuli objects were presented at the four corners and the center of the screen. Each object was framed in a square with a diagonal of 6.5 cm.

**Figure 2:**
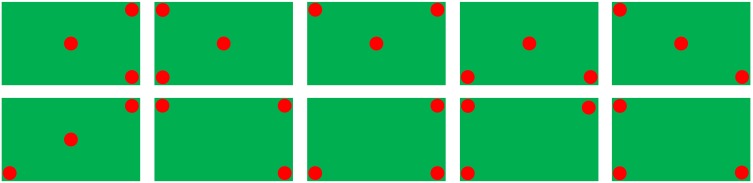
**Ten types of target patterns (a dot indicates an object)**.

Four shapes (including circles, horizontal stripes, triangles, and vertical stripes) in seven different colors (including black, blue, brown, green, red, white, and yellow) were used as stimuli objects. The combinations of all different stimuli objects formed two types of object sets: target and non-target. For an object set to be considered as a target, three out of five objects must be the same in both color and shape (e.g., Figure 1A); otherwise, it was a non-target (e.g., Figure 1B). Figure 2 (where a dot represented an object in any shape and color) illustrates all of the 10 types of target patterns, which covered different visual fields.

The peripheral visual task lasted for two test sessions and each test session had 56 trials. Session 1 included 13 target trials and 43 non-target trials, and all objects were circles but in different colors. Session 2 comprised 14 target trials and 42 non-target trials, and it covered all situations in different colors and shapes. The sequence and exposure time of the stimuli trials was determined by a script file which was programmed and loaded into the system before the experiment started, and there was no interval or cue between consecutive trials. In both test sessions, the exposure time of trials progressively decreased from 4 to 0.5 s. More specifically, the exposure time was 4 s for Trial 1 ~ Trial 7, 3 s for Trial 8 ~ Trial 14, 2 s for Trial 15 ~ Trial 22, 1.5 s for Trial 23 ~ Trial 30, 1 s for Trial 31 ~ Trial 38, and 0.5 s for Trial 39 ~ Trial 56, respectively.

The subjects were seated on a comfortable chair with adjustable height to keep their eyes centered on the screen and their eyes 53 cm away from the screen. The distance of 53 cm ensured that the full horizontal vision angle and vertical vision angle were 75.18° and 60° respectively. To start each test session, the start button in the upper left corner was clicked by the subjects with eye scanning. Then the subjects needed to look back to the central object. The test started after 2 s of the click. During the test, the central object kept random movement within −10% to 10% screen size from the center every 10 ms, with a movement step of 0 to 1% of the screen size. The subjects were required to track the central moving object binocularly with a mouse pointer and keep their sight on the central object at all times so that they could capture the corner objects with their peripheral vision. Once the subjects perceived the target pattern, they needed to click on the central object as fast as possible. They were not allowed to use eye scanning, although most of the subjects did use it voluntarily or involuntarily.

### Eye scanning detection

The aim of the detection algorithm was to detect large eye movements which occurred in perceiving the four corner objects of the screen. The eye scanning during visual task was determined by the information presented in the two EOG channels. The direction of the eye movement was not considered in the eye scanning detection algorithm, i.e., no distinction between left, right, up, or down eye movement. The small eye movements could be produced when tracking the central moving object binocularly. In order to achieve the compromise between the detection of large eye movements and reduction of the false positive (FP) rate due to the small eye movement in the tracking and the influence of other artifacts, the following EOG detection algorithm was adopted.

The signal when the subject clicked the start button was taken as the calibration sample. The calibration sample and the test signal were filtered by a low pass filter with a 5 Hz cut off frequency. Then, the absolute maximum of the calibration sample was determined and the test signal was normalized by this value. The signal extremes were found when the first derivative of the signal was zero. Only the extremes with absolute value higher than 60% of that absolute maximum were considered as possible candidates for EOG. In most cases this was enough to conclude that EOG presented in the channel.

By this algorithm, it was possible to detect if the subject employed only peripheral vision or helped by eye scanning to perceive the four corners of the screen. If an EOG was detected after the beginning of a stimulus set and before the subject's response, it was considered as eye scanning (Rodrigues et al., 2012).

### Data analysis

#### Peripheral visual performance

The dynamic peripheral visual performance was described by the accuracy. Regarding each test session, the following events were taken into account: true positive (TP) meant clicking a target; true negative (TN) stood for ignoring a non-target; FP was accounted when a non-target was clicked; false negative (FN) stood for ignoring a target. The above events were employed to calculate the accuracy shown in Equation (1), where T was the total number of targets and NT was the total number of non-targets. If the subject did not click on any target or clicked on all (true and false targets), the accuracy was 0%. If the subject only clicked on correct targets and did not miss any one the accuracy was 100%. If the subject clicked on every false target and did not click on any correct one the accuracy was −100%.

(1) $$\text{accuracy}=\frac{1}{2}\left[\left(\frac{\text{TP}}{\text{T}}-\frac{\text{FP}}{\text{NT}}\right)+\left(\frac{\text{TN}}{\text{NT}}-\frac{\text{FN}}{\text{T}}\right)\right]\times 100\%$$

#### Alpha activity

**Figure 3:**
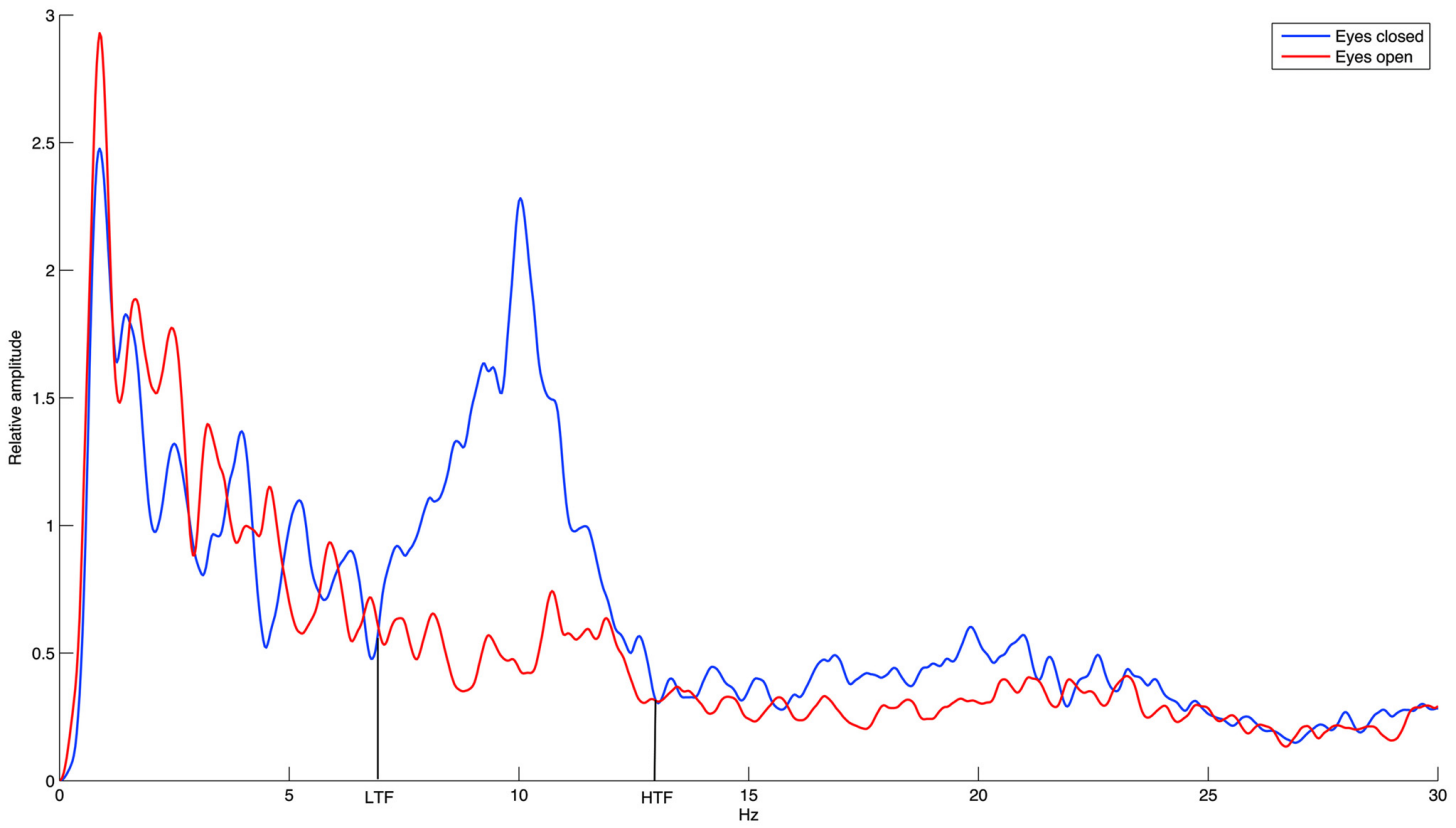
**The demonstration of individual alpha band (IAB)**.

Considering the large inter-individual difference in the alpha band, we calculated the relative alpha amplitude not only in the standard alpha band (8–12 Hz) but also in the individual alpha band (IAB) determined by the amplitude spectrum crossings between the eyes-open resting baseline and the eyes-closed resting baseline (Klimesch, 1999). As demonstrated in Figure 3, the IAB ranged from the low transition frequency (LTF) to the high transition frequency (HTF).

The relative amplitude during each recording was calculated using Equation (2), where *k* was an index over frequency and *X*(*k*) was the frequency amplitude spectrum calculated by fast Fourier transformation (FFT) using all sample points in the 1-min eyes-closed resting recording, 1-min eyes-open resting recording, and in each stimuli trial during the peripheral vision task. The average amplitude of all stimuli trials was taken as the relative amplitude during the peripheral vision task.

(2) $$\text{relative amplitude}=\frac{\frac{\sum_{k\;=\;LTF}^{HTF}X(k)}{HTF-LTF}}{\frac{\sum_{k\;=\;0.5}^{30}X(k)}{30-0.5}}$$

#### Statistical analysis

Firstly, the data distribution was examined by the one-sample Kolmogorov–Smirnov test. All data were found normally distributed. However, there were outliers in the alpha amplitude detected using the adjusted boxplot rule (Pernet et al., 2013). Pearson correlation analysis is particularly sensitive to outliers, which can distort coefficients relative to their true values (Wilcox, 2001, 2012), while percentage-bend correlation analysis protects against univariate outliers (Pernet et al., 2013). Therefore, percentage-bend correlation was adopted to examine the inter-individual correlations between the dynamic peripheral visual performance and the alpha activity in the eyes-open resting baseline, eyes-closed resting baseline, and peripheral vision task, respectively.

## Results

**Figure 4:**
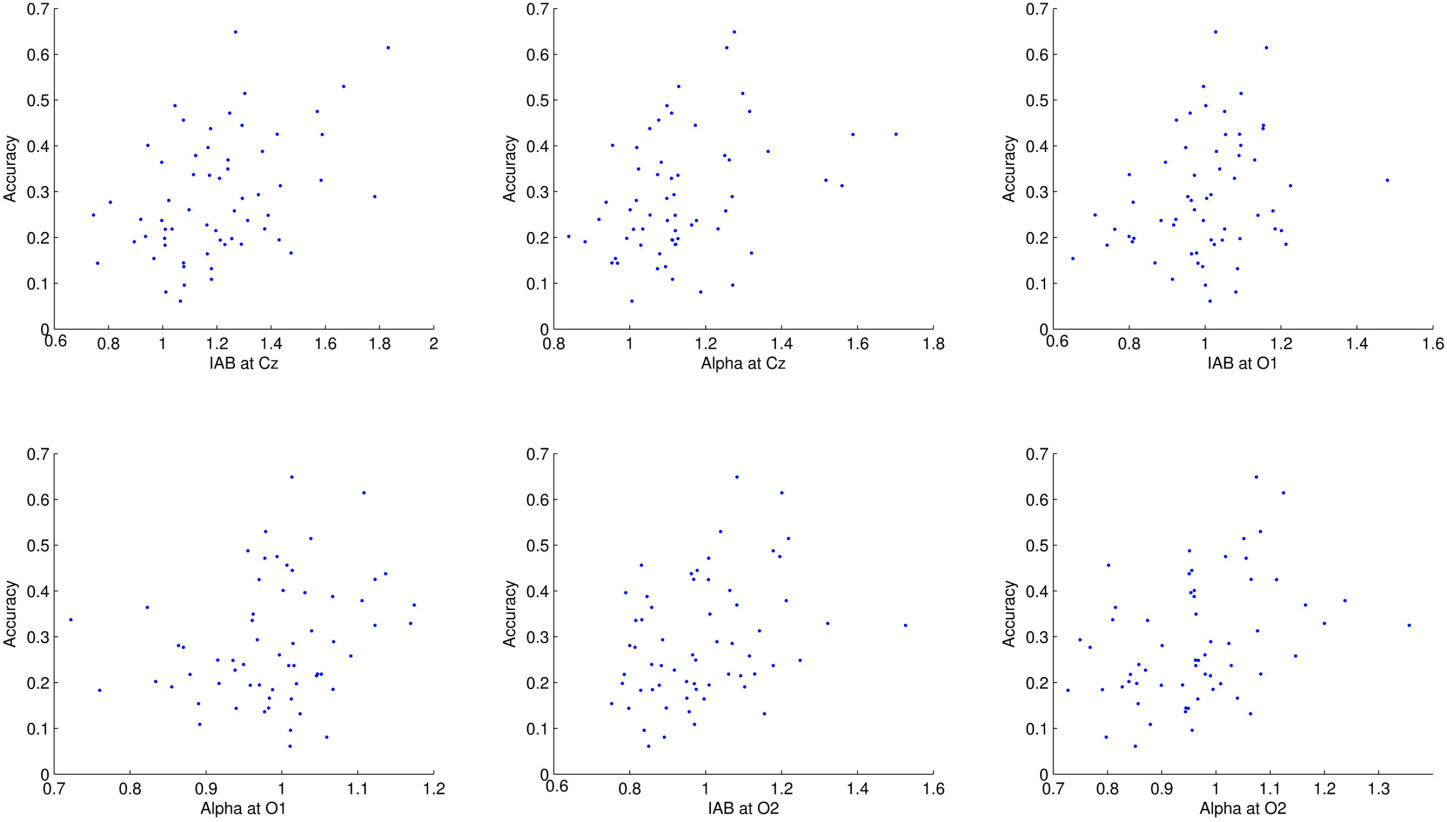
**Scatterplots of dynamic peripheral visual performance with alpha activity during visual task**. Each dot corresponds to one subject.

The average dynamic peripheral visual performance was 28.72% (*SD* = 13.18%) across all subjects, ranging from 6.11 to 64.88%. Table 1 presents the mean and *SD* of alpha amplitude in the resting condition and during the visual task. Table 2 presents the percentage-bend correlation coefficients and 95% confidence interval (CI) after bootstrapping between the dynamic peripheral visual performance and the alpha activity in all conditions. As shown in the Table 2, the dynamic peripheral visual performance had no significant correlation with the alpha activity in the eyes-open and eyes-closed resting conditions. However, it is not the case for the alpha activity during the visual task. The dynamic peripheral visual performance had significant positive correlations with the amplitudes of alpha (8–12 Hz) and IAB at all three locations. Figure 4 presents the scatterplots of the dynamic peripheral visual performance and the alpha activity during visual task.

**Table 1:** **Mean ± *SD* of alpha amplitude in the resting condition and during the visual task**.

**EEG amplitude**	**Location**	**In resting with eyes open**	**In resting with eyes closed**	**During peripheral vision task**
IAB amplitude	Cz	1.286 ± 0.355	1.826 ± 0.412	1.199 ± 0.229
	O1	1.182 ± 0.384	1.919 ± 0.551	1.003 ± 0.142
	O2	1.225 ± 0.384	1.950 ± 0.515	0.990 ± 0.155
Alpha amplitude	Cz	1.277 ± 0.338	1.739 ± 0.371	1.138 ± 0.166
	O1	1.186 ± 0.328	1.844 ± 0.517	0.988 ± 0.090
	O2	1.224 ± 0.360	1.900 ± 0.505	0.965 ± 0.124

**Table 2:** **Correlation coefficients between the dynamic peripheral visual performance and the alpha activity**.

**Condition**	**Location**	**95% CI after bootstrapping**		**Correlation coefficients**	
		**Alpha**	**IAB**	**Alpha**	**IAB**
In resting with eyes open	Cz	[−0.257, 0.258]	[−0.247, 0.270]	−0.003	0.004
	O1	[−0.345, 0.128]	[−0.333, 0.180]	−0.089	−0.105
	O2	[−0.262, 0.260]	[−0.258, 0.251]	−0.022	0.003
In resting with eyes closed	Cz	[−0.250, 0.288]	[−0.217, 0.246]	0.028	0.015
	O1	[−0.235, 0.299]	[−0.303, 0.236]	0.0296	−0.034
	O2	[−0.229, 0.291]	[−0.199, 0.292]	0.054	0.060
During peripheral vision task	Cz	[0.105, 0.562]	[0.143, 0.570]	0.348^**^	0.361^**^
	O1	[0.043, 0.489]	[0.063, 0.461]	0.290^*^	0.265^*^
	O2	[0.147, 0.575]	[0.045, 0.515]	0.370^**^	0.302^*^

* p < 0.05;

** p < 0.01.

## Discussion

The dynamic peripheral visual ability is important for all people in the real visual world. This work aimed to investigate the relationship between the dynamic peripheral visual performance and the alpha activity. The results demonstrated that the dynamic peripheral visual performance positively correlated with the amplitudes of alpha and IAB *during* the peripheral vision task, but no correlation in the eyes-open and eyes-closed resting conditions.

Some studies have demonstrated the relationship between the perception performance in the central vision and the resting alpha activity. Romei et al. (2008) reported an inverse relationship between the resting alpha power and the visual cortex excitability across subjects, i.e., a decrease in the cortical excitability with increasing posterior alpha power. Moreover, Hanslmayr et al. (2005, 2007) also found a negative inter-individual correlation between the resting alpha amplitude and the central vision perception performance. In our case, we investigated the dynamic peripheral vision rather than the central vision and we did not find out any relation between the resting alpha activity and the dynamic peripheral visual performance. Such a difference between this study and previous studies might be due to the reasons as follows.

A major reason is the visual task difference. In Romei et al. (2008), transcranial magnetic stimulation (TMS) stimulated early visual cortex (V1/V2) and bypassed the peripheral visual pathway, which permitted to obtain a direct measure of cortical excitability. Romei et al. (2008) measured the cortical excitability which was different from the dynamic peripheral vision ability, thus it is not comparable between Romei's work and this study. In the visual discrimination task from Hanslmayr et al. (2005, 2007), two or four letters were presented very briefly in the center of the screen and the subjects were instructed to indicate which letter they perceived by pressing the buttons. Whereas in the dynamic peripheral vision task, the central object kept random movement in a small range, and the subjects were instructed to track the central moving object binocularly with a mouse pointer and keep their sight on the central moving object meanwhile perceive four corner objects with their peripheral vision. Once they perceived the target (i.e., three out of five objects were exactly the same), they needed to click on the central moving object as fast as possible. It is obvious that the central vision task and the dynamic peripheral vision task are totally different.

Another important reason lies in the difference of physiological mechanisms of visual processing in central and peripheral vision. Firstly, there are neuroanatomical differences between central and peripheral visions. The central vision is represented over a larger fraction of cortical surface than a comparable extent of the peripheral visual field (Wandell et al., 2007) and more neural tissue is dedicated to processing centrally presented stimuli than peripherally presented stimuli (Brown et al., 2005). Moreover, reaching in foveal and extrafoveal vision depend on two different neural substrates. Prado et al. (2005) reported that reaching to an object in central vision involved a restricted network, including the medial intraparietal sulcus (mIPS) and the caudal part of the dorsal premotor cortex (PMd). Reaching to an object in peripheral vision activated in addition the parieto-occipital junction (POJ) and a more rostral part of PMd. Thus, reaching to the peripheral visual field engages a more extensive cortical network than reaching to the central visual field (Prado et al., 2005). Finally, anatomical and electrophysiological studies of the extrastriate cortex suggest that visual processing in the far peripheral visual field is likely to involve a distinct network of specialized cortical areas, located in the depths of the calcarine sulcus and interhemispheric fissure (Hu et al., 2014).

This study found significant positive correlations between the dynamic peripheral visual ability and the alpha activities during the peripheral visual task, indicating that a subject with higher alpha during the visual task had better peripheral visual ability. Klimesch et al. (2007) concluded that alpha synchronization plays an active role for the inhibitory control and timing of cortical processing. However, Palva and Palva (2007) argued that it is unfeasible to deduce in a one-track fashion that large alpha amplitudes correspond to inhibited or disengaged cortical states. In our case, a greater level of alpha amplitude during the peripheral vision task may reflect the inhibition of non-essential activity, which in turn may facilitate performance on the peripheral vision task.

Previous studies have also demonstrated that the prestimulus alpha is related to central vision perception performance. Reduced prestimulus alpha power improves the central vision detection performance of near threshold stimuli or more veridical perception in visual discrimination tasks (Ergenoglu et al., 2004; Hanslmayr et al., 2007; van Dijk et al., 2008; Mathewson et al., 2009; Wyart and Tallon-Baudry, 2009). Moreover, the prestimulus posterior alpha rhythm is actively involved in shaping forthcoming perception and constitutes a substrate rather than a mere correlate of visual input regulation (Romei et al., 2010). However, Lange et al. (2013) demonstrated that reduced prestimulus occipital alpha power enhanced excitability of visual cortex rather than improved visual perception. On the other hand, Klimesch et al. (2011) emphasized that the type of event-related alpha power changes (as measured by event-related desynchronization/synchronization or ERD/ERS) as well as the time course of a power change (associated with ERD or ERS) in visual stimulation are strictly task-dependent. Specifically, ERD may precede visual stimulation (Klimesch et al., 1998), may occur immediately (Woertz et al., 2004), or may be delayed (Klimesch et al., 2000). In some cases, visual stimulation even elicits ERS (Klimesch, 1999). Regarding the dynamic peripheral vision, the roles of prestimulus alpha and alpha ERD/ERS are unknown, which can be explored in future studies for a deeper understanding of the dynamic peripheral vision mechanism. To achieve these goals, the prestimulus and poststimulus measurement should be added in each stimuli trial in the peripheral vision task. Moreover, the event related alpha power changes in target pattern and non-target pattern can be analyzed in the time periods between the prestimulus and the beginning of each trial, as well as between the prestimulus and poststimulus, respectively. Besides the EEG study, the cortical networks of the peripheral visual field require further research attention, which currently is poorly understood (Hu et al., 2014).

To our knowledge, this is the first study to investigate the relationship between the dynamic peripheral visual performance and the alpha activity. We found that the dynamic peripheral visual performance had significant positive inter-individual correlations with amplitudes in alpha band and IAB *during* the peripheral vision task. This finding provides hints for peripheral vision performance enhancement by regulating alpha activity using neuromodulation techniques. Based on the findings, a recent attempt succeeded in improving the dynamic peripheral vision performance in normal people by up-regulating alpha amplitude using neurofeedback (Nan et al., 2013).

### Conflict of interest statement

The authors declare that the research was conducted in the absence of any commercial or financial relationships that could be construed as a potential conflict of interest.
